# *In vivo* magnetic resonance imaging of the human limbic white matter

**DOI:** 10.3389/fnagi.2014.00321

**Published:** 2014-11-27

**Authors:** Susumu Mori, Manisha Aggarwal

**Affiliations:** ^1^Russell H. Morgan Department of Radiology and Radiological Science, Johns Hopkins University School of MedicineBaltimore, MD, USA; ^2^F.M. Kirby Research Center for Functional Brain Imaging, Kennedy Krieger InstituteBaltimore, MD, USA

**Keywords:** limbic system, MRI, diffusion, human, white matter, *in vivo*

## Abstract

The limbic system mediates memory, behavior, and emotional output in the human brain, and is implicated in the pathology of Alzheimer’s disease and a wide spectrum of related neurological disorders. *In vivo* magnetic resonance imaging (MRI) of structural components comprising the limbic system and their interconnections via white matter pathways in the human brain has helped define current understanding of the limbic model based on the classical circuit proposed by Papez. MRI techniques, including diffusion MR imaging, provide a non-invasive method to characterize white matter tracts of the limbic system, and investigate pathological changes that affect these pathways in clinical settings. This review focuses on delineation of the anatomy of major limbic tracts in the human brain, namely, the cingulum, the fornix and fimbria, and the stria terminalis, based on *in vivo* MRI contrasts. The detailed morphology and intricate trajectories of these pathways that can be identified using relaxometry-based and diffusion-weighted MRI provide an important anatomical reference for evaluation of clinical disorders commonly associated with limbic pathology.

## Introduction

The limbic system consists of a group of interconnected nuclei and cortical structures in the brain that mediate emotion, memory, and behavior (Patestas and Gartner, 2006). The classical circuit described by Papez includes important white matter pathways interlinking the hippocampus, mammillary bodies, anterior thalamic nuclei, cingulate gyrus (Cg), and the parahippocampal gyrus, that form a closed loop in each hemisphere (Papez, 1995). The complex connections of the limbic system are implicated in a wide array of neurological disorders including Alzheimer’s disease, mild cognitive impairment, temporal lobe epilepsy, and schizophrenia, and continue to be defined using functional neuroimaging and magnetic resonance imaging (MRI) studies (Naidich et al., 1987a,b; Mark et al., 1995; Concha et al., 2010; Catani et al., 2013). This purpose of this review is to focus on the use of MRI methods for delineation of some of the major white matter tracts that are associated with the limbic system.

White matter tracts in the human brain can be broadly classified into three categories; association, projection, and commissural tracts (Nieuwenhuys et al., 2008). Association tracts establish connections across different cortical regions within the same hemisphere. Short association fibers form connections between different gyri within the same lobe, while long association fibers form intra-hemispheric connections across different lobes. Commissural tracts also establish connections between different cortical areas, but these specifically refer to inter-hemispheric connections. The projection tracts connect the cortex to other parts of the brain, such as deep nuclei, brain stem, cerebellum, and spinal cord. The tracts that interconnect the gray matter structures of the limbic system could be mainly categorized as projection or association tracts under this system of classification.

Advanced MRI techniques, particularly diffusion MRI, have afforded significant insights into the anatomy of the limbic system in the human brain, by enabling non-invasive and three-dimensional mapping of structural connectivity *in vivo*. Both gray and white matter components of the limbic system have been studied by using relaxation-based (T1- or T2-weighted) MRI in Alzheimer’s disease (Smith et al., 1999; Callen et al., 2001) as well as other neurological disorders affecting the limbic system (Atlas et al., 1986; Ng et al., 1997; Kuzniecky et al., 1999; Oikawa et al., 2001; Tsivilis et al., 2008; Lövblad et al., 2014). Diffusion MRI, which is based on sensitization to the directional-dependence of NMR signal attenuation arising from restricted diffusion of water molecules in brain tissue (Moseley et al., 1990; Le Bihan, 2003), has been used to investigate the structural connectivity of white matter tracts in the limbic system (Yamada et al., 1998; Wakana et al., 2004; Concha et al., 2005; Kalus et al., 2006; Malykhin et al., 2008; Zeineh et al., 2012), as well as to examine limbic pathways under pathologic conditions (Haznedar et al., 2000; Hattingen et al., 2007; Dineen et al., 2009; Wilde et al., 2010). Diffusion MRI employs a pair of diffusion-weighting gradients in the MR pulse sequence to impart sensitization to water diffusion in the brain which is influenced by surrounding tissue microstructure. Diffusion tensor imaging (DTI) uses a Gaussian approximation of diffusion and is based on fitting the resulting signal decay to a six-element tensor model to estimate the degree and orientation of diffusion anisotropy, which can provide *in vivo* estimates and specific quantitative measures of white matter fiber structure and orientation (Basser and Jones, 2002; Tournier et al., 2011).

**Figure 1:**
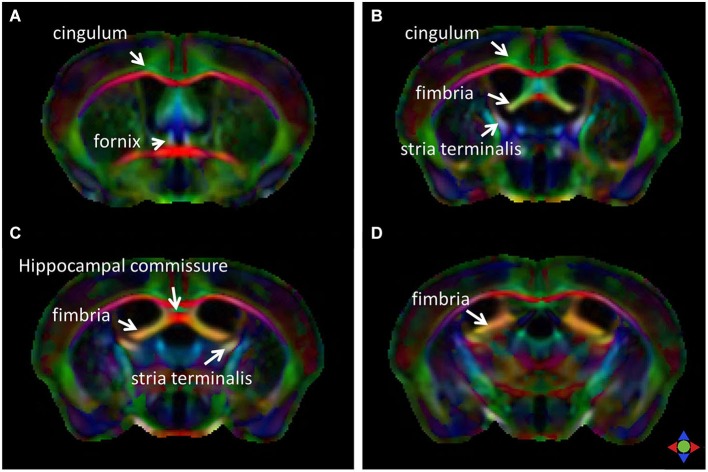
**Major limbic tracts in the mouse brain identified with diffusion tensor imaging (DTI)**. Direction-encoded color (DEC) maps derived from DTI in four coronal sections (**A–D**, from rostral to caudal) demonstrate the limbic tracts delineated on the basis of the primary orientation of diffusion anisotropy. Red, green, and blue in the color maps denote diffusion along the medial-lateral, anterior-posterior, and dorsal-ventral axes, respectively.

**Figure 2:**
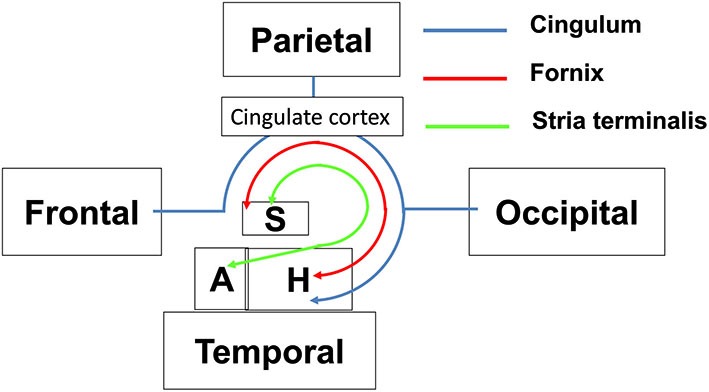
**Schematic diagram illustrating the structural connectivity in the limbic system via major limbic tracts in the human brain**. Structural abbreviations are: A: amygdala, H: hippocampal formation, S: septum.

**Figure 3:**
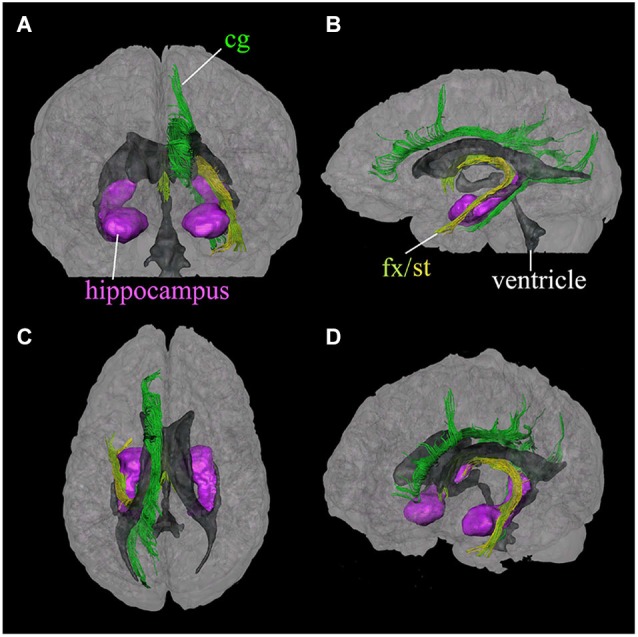
**Three-dimensional reconstruction of limbic system tracts in the human brain based on DTI**. Reconstructed tracts in four different viewing angles through the brain are shown; **(A)** anterior view, **(B)** left lateral view, **(C)** superior view, and **(D)** oblique view from a right anterior angle. Reconstructed fibers are; cingulum (cg, dark green), fornix (fx, light green), and stria terminalis (st, yellow). The hippocampus and amygdala (purple) and the ventricles (gray) are shown for anatomical reference. (Reproduced with permission from Wakana et al. (2004)).

The limbic tracts in the human brain are relatively small in comparison to other mammalian species. For instance, major limbic tracts that can be identified with DTI in a mouse brain are demonstrated in Figure 1. It can be seen that these limbic tracts are large and well-defined, with the exception of the cingulum, which is relatively diffuse and characterized by an ambiguously defined boundary. As will be examined in detail in the following sections, many of these tracts are comparatively difficult to identify in the human brain. Interestingly, one exception is the cingulum, which is one of the most readily identifiable tracts in the human white matter (Burgel et al., 2006). Figure 2 illustrates a connectivity diagram of the three major limbic tracts that are identifiable, albeit partially, in the human brain; namely, the cingulum, the stria terminalis, and the fornix. The Cg, which constitutes a part of the limbic system, receives sensory inputs from the neocortex (frontal, parietal, occipital, and temporal lobes) and projects to the hippocampal complex via the cingulum bundle. Anatomically, the cingulum forms a large outer C-shaped loop. The fornix also has a C-shaped trajectory that is nested within the cingulum, which is known to contain bidirectional connections between the septal area and the hippocampus. The stria terminalis forms the inner-most C-shaped trajectory connecting the septal area and the amygdala. The three-dimensional reconstruction of these tracts based on deterministic fiber tractography from a human DTI study is shown in Figure 3. The cingulum can be delineated coursing along the ventral surface of the hippocampal formation, while the fornix and stria terminalis project primarily along its dorsal surface (Figure 3).

## MR imaging of limbic tracts in the human brain

Relaxometry-based and diffusion-weighted MR acquisitions generate complementary tissue contrasts for examination of limbic white matter anatomy, as will be shown in the following sections. T1-weighted imaging generally offers higher spatial resolution due to its relatively high signal-to-noise efficiency within clinically viable scan times. This can be specifically advantageous for visualizing the detailed morphology of the often highly convoluted limbic structures, such as the Cg and the hippocampal formation. One drawback of relaxation-based MRI is the lack of high anatomical contrast to decipher the architecture of white matter structures and tracts in the limbic system. On the other hand, DTI can provide rich tissue contrast for visualizing white matter axonal architecture, based on the orientation of structural barriers (e.g., axonal membranes and myelin sheaths) that restrict water diffusion preferentially along directions orthogonal to the long axis of the axons. DTI, however, is limited in terms of the achievable spatial resolution *in vivo*. The inherent limitation on the spatial resolution for DTI stems from its high sensitivity to physiological motion and the ensuing necessity to use single-shot rapid imaging—constraints which can be circumvented to some extent for *ex vivo* DTI studies, thereby allowing higher spatial resolution acquisitions as shown in Figure 1. In the following sections, the anatomy of major limbic tracts delineated with MR studies of the human brain *in vivo* will be described in detail.

### Cingulum bundle

**Figure 4:**
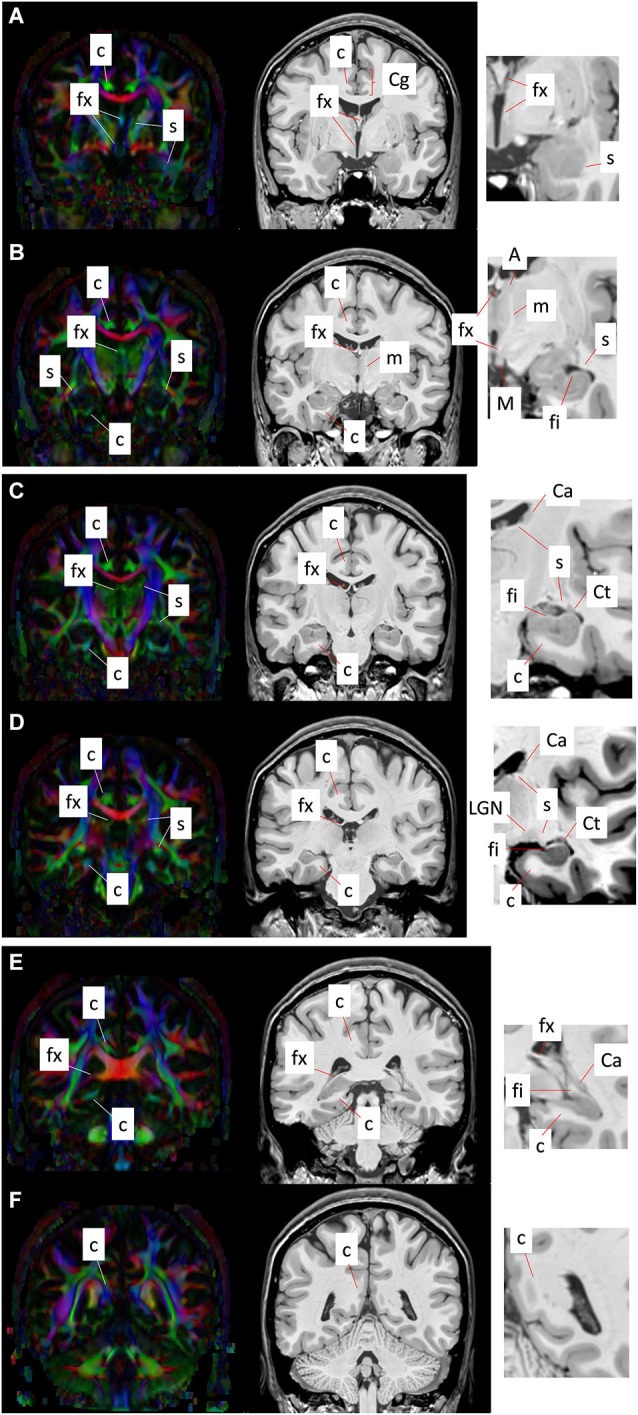
**Coronal T1-weighted images (right panel) and corresponding DEC maps derived from DTI (left panel) showing major limbic structures and tracts delineated with MRI contrasts**. Images **(A–F)** show six coronal sections from the anterior to the posterior direction. Red, green, and blue in the DEC maps represent the primary orientation of diffusion along the medial-lateral, anterior-posterior, and superior-inferior axes, respectively. High-magnification views of select regions from T1-weighted contrasts are shown at the right, indicating the gray and white matter limbic structures that can be identified using MRI. Structural abbreviations are; c: cingulum, Ca: caudate, Cg: cingulate gyrus, Ct: tail of the caudate, fi: fimbria, fx: fornix, LGN: lateral geniculate nucleus, m: mammillothalamic tract, M: mammillary body, s: stria terminalis.

Figure 4 shows a series of coronal sections from high-resolution T1-weighted imaging and DTI of the human brain. The T1-weighted images shown are acquired on a 7T MR scanner, using a magnetization-prepared rapidly-acquired gradient echo (MPRAGE) sequence with whole brain coverage and a matrix size of 368 × 368 × 261, resulting in an isotropic spatial resolution of 0.625 mm. The DTI data are acquired at 3T using a single-shot echo planer imaging (EPI) sequence, with matrix size of 128 × 128 × 72 and isotropic spatial resolution of 1.8 mm. The *b*-value for DTI was 1000 s/mm^2^, with diffusion encoding applied along 32 non-collinear gradient directions.

The cingulum constitutes a compact bundle of both long and short association fibers that connect the cingulate cortex to the parahippocampal gyrus, prefrontal cortex, and cortical association areas in the parietal and occipital lobes (Schmahmann et al., 2007; Nieuwenhuys et al., 2008; Nezamzadeh et al., 2010). Delineation of the cingulum bundle based on MR contrasts across coronal sections from anterior to posterior (Figures 4A–F) can be seen in Figure 4. The cingulum can be readily identified in the direction-encoded color (DEC) maps in Figures 4A–D, marked by a distinct anterior-posterior orientation (green in DEC maps) adjacent to the cortical gray matter in the Cg and the corpus callosum. It makes an almost 180° U-turn ventrally around the splenium of the corpus callosum toward the temporal lobe, and as it turns to the superior-inferior direction, its color changes to blue in DEC contrasts (Figure 4F). After curving around the splenium, it projects along the inferior surface of the hippocampus and becomes smaller and more diffuse towards the anterior pole of the hippocampus (Figures 4A–D). The projection in the temporal lobe can also be appreciated in DEC maps in Figures 4B–D. The location of the cingulum can be estimated from corresponding T1-weighted images in coronal views (Figures 4A–F), however its boundary is less obvious and cannot be clearly distinguished from neighboring white matter tracts in T1-weighted contrasts.

### Fornix

**Figure 5:**
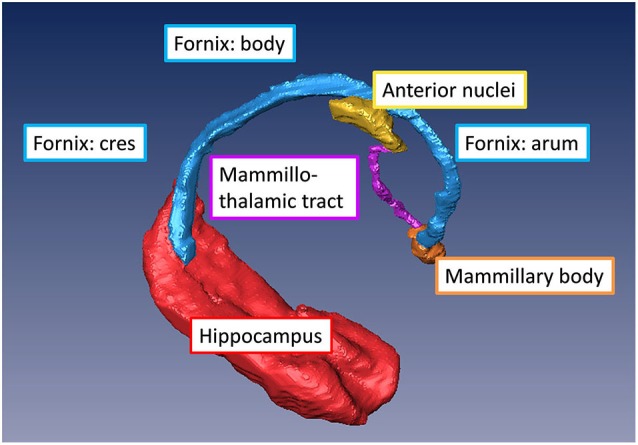
**Major gray and white matter limbic structures showing efferent connections of the hippocampal formation to the mammillary body (via the fornix) and to the anterior thalamic nuclei (via the mammillothalamic tract) reconstructed from *in vivo* T1-weighted MRI of the human brain**.

The fornix is a projection tract that constitutes the major efferent fiber pathway from the hippocampal region, connecting it with the mammillary body, and then to the anterior thalamic nuclei through the mammillothalamic tract (Nolte, 1998; Nieuwenhuys et al., 2008; Thomas et al., 2011). This part of the Papez circuit, as well as the participating gray matter structures, can be precisely reconstructed from the high-resolution T1-weighted MR images as shown in Figure 5. The anatomical locations of these structures can also be appreciated in the coronal sections of T1-weighted images shown in Figures 4A–F. Fibers in the fornix arise from the hippocampus in each hemisphere, continue into the fimbria (delineated in T1-weighted contrasts in Figures 4C,D) and form the crus of the ipsilateral fornix. The crura continue forward and converge under the splenium of the corpus callosum to form the body of the fornix. Fimbrial fibers that continue medially across the midline to the contralateral hemisphere form the commissural component of the fornix known as the hippocampal commissure, which projects to the contralateral hippocampus, and is relatively less distinct in MR contrasts in the human brain in contrast to its large size and prominent delineation in mouse brains (Figure 1). Because of the relatively small size of this fine white matter tract, DTI contrasts can reveal only parts of the fornix in the brain *in vivo* (DEC maps in Figure 4). Several studies have examined pathological changes in the fornix associated with Alzheimer’s disease (Oishi et al., 2012; Fletcher et al., 2013, 2014) and related disorders (Ng et al., 1997; Kuzniecky et al., 1999) using MRI. While the body and crus of the fornix are more apparent in T1-weighted and DEC contrasts, the anterior portion of the fornix as it approaches the septal and hypothalamic regions becomes more diffuse and narrow. Although there are several different anatomical targets of fibers in the fornix in this region, only the projection towards the mammillary body can be clearly delineated in high-quality T1-weighted images (Figure 4).

### Stria terminalis/fimbria

The stria terminalis is a limbic pathway that constitutes the major efferent connection from the amygdala to the septal nuclei and the hypothalamus (Parent, 1996). Because of its small size, the stria terminalis is relatively difficult to recognize in both T1-weighted and DTI contrasts, but can be identified sporadically at several locations. Along the majority of its trajectory, it travels adjacent to the medial surface of the caudate nucleus, all the way to the tip of the tail of the caudate as can be clearly seen in DTI contrasts in Figures 4C–E. Here again, T1-weighted images provide an estimate of the location of the stria terminalis in coronal sections, but its precise boundary cannot be demarcated due to the lack of clear tissue contrast within the white matter in T1-weighted MRI. The ventral portion of the stria terminalis is relatively difficult to observe with MRI. Interestingly, as the stria terminalis travels dorsally, along the roof of the inferior horn of the lateral ventricles, there is a region where the DTI contrast intensifies with a marked increase in fractional anisotropy (Figure 4D). Because this region is adjacent to the lateral geniculate nucleus (LGN), it is possible that fibers in this region are mixed with adjacent fibers from the optic radiation. The fimbria travels primarily along the dorsal surface of the hippocampus, which can be clearly identified in the high-resolution T1-weighted images (Figures 4B–D). Although the stria terminalis and the fimbria are anatomically separated along opposite banks of the inferior horn of the ventricles, it is difficult to distinguish them in this region with DTI, owing to limited spatial resolution and resulting partial volume effects in *in vivo* diffusion-weighted images. The stria terminalis continues to travel anteriorly, finally reaching the amygdaloid complex (Figures 4A,B). Although small, this section of the stria terminalis, not contaminated by adjacent fibers from the fimbria and optic radiation, can be clearly identified in high-quality DTI data (Figures 4A,B).

## Conclusion and future directions

In this mini review, we have described major tracts of the limbic system that can be delineated based on *in vivo* MRI of the human brain at clinical gradient strengths. The intricate three-dimensional morphologies of gray and white matter structures and interconnecting pathways of the limbic circuitry that can be resolved using non-invasive MR methods are important for clinical studies and for evaluation of various neurologic disorders that affect the limbic system. Functional and structural neuroimaging studies continue to refine existing conception of the limbic system and its disorders. Advances in both MR hardware, including higher field strengths (7T–9.4T) and high-performance gradient systems (e.g., 300 mT/m) for human scanners, as well as diffusion MR acquisition and modeling techniques, will be instrumental in pushing the envelope of the achievable resolution and the level of structural detail for *in vivo* studies of the human brain, which can potentially afford additional insights into deciphering the complex circuitry of the limbic system.

## Conflict of interest statement

The authors declare that the research was conducted in the absence of any commercial or financial relationships that could be construed as a potential conflict of interest.
